# Asymmetric Dynamics Between the Protomers of the σ2 Receptor Homodimer

**DOI:** 10.1021/acs.jcim.5c02174

**Published:** 2025-11-06

**Authors:** Manming Xu, Saleh Alyemni, Veniamin A. Borin, Ranabir Majumder, Nathaniel V Nucci, Thomas Keck, Kevin Frankowski, Pratul K. Agarwal, Shozeb Haider

**Affiliations:** 1 371646 UCL School of Pharmacy, London WC1N 1AX, U., KUK; 2 Department of Physiological Sciences, 7618 Oklahoma State University, Stillwater, Oklahoma 74078, United States; 3 Department of Biological and Biomedical Sciences, 3536 Rowan University, Glassboro, New Jersey 08028, United States; 4 Department of Physics and Astronomy, 3536 Rowan University, Glassboro, New Jersey 08028, United States; 5 Department of Chemistry and Biochemistry, 3536 Rowan University, Glassboro, New Jersey 08028, United States; 6 Center for Integrative Chemical Biology and Drug Discovery, Eshelman School of Pharmacy, 2331 University of North Carolina, Chapel Hill, North Carolina 27599, United States; 7 High-Performance Computing Center, 7618 Oklahoma State University, Stillwater, Oklahoma 74078, United States; 8 University of Tabuk (PFSCBR), Tabuk 71491, Saudi Arabia; 9 UCL Centre for Advanced Research Computing, London WC1H 9RL, UK

## Abstract

The sigma-2 receptor (σ_2_R/TMEM97) is a clinically relevant membrane protein involved in cholesterol regulation and overexpressed in cancer and neurodegenerative diseases. Despite its therapeutic potential, the dynamic mechanisms underlying σ_2_R function and ligand binding remain poorly understood. Here, we combined adaptive sampling molecular dynamics simulations with quasi-anharmonic analysis and unsupervised machine learning method to investigate the conformational behavior of the σ_2_R homodimer in both apo and cholesterol-bound states. Our results reveal asymmetric dynamics between the two protomers. This asymmetry is driven by anticorrelated helical motions and mutually exclusive salt bridge formation, including a switching mechanism between K_55_–E_139_ and D_122_–R_140_. Cholesterol binding further enhances this asymmetry by stabilizing one protomer and altering the dynamics of the other. Species-specific allosteric interaction between D_56_–R_133_ may be essential for the human σ_2_R function. Additional lipid–protein interaction analysis highlights asymmetric membrane coupling in the bound state. These findings provide a plausible explanation for the receptor’s dimeric nature, suggesting that ligand binding at one site may allosterically influence the apo protomer, thereby modulating receptor function. Our work provides new mechanistic insight into σ_2_R function and highlights the importance of asymmetric dynamics.

## Graphical abstract

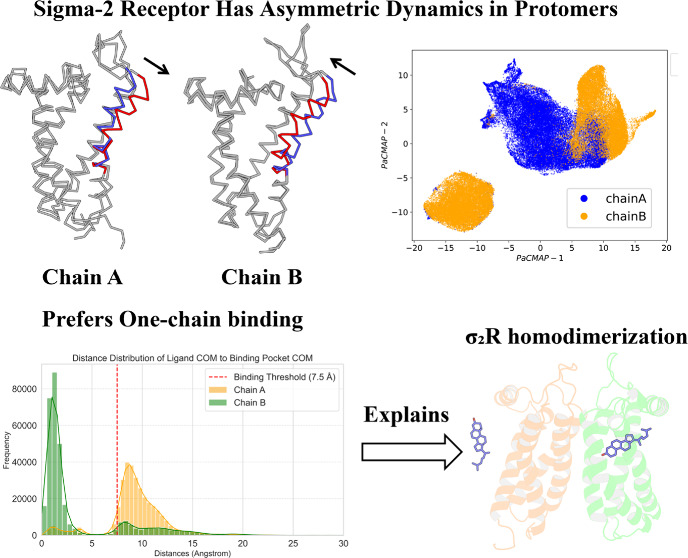

## Introduction

Sigma receptors (σRs) are regulatory proteins involved in various homeostatic processes, including the modulation of ion channels,^1^ regulation of cellular cholesterol levels,^2^ and control of cell proliferation and apoptosis.^3^ This receptor family comprises of two genetically unrelated subtypes: the sigma-1 receptor (σ_1_R) and the sigma-2 receptor (σ_2_R).^4^ The σ_2_R, in particular, has attracted increasing attention for its role in cholesterol metabolism, its overexpression in cancer cells,^5^ and its potential as a therapeutic target in Alzheimer’s disease.^6^

It is now well recognized that σ_2_R functions by forming protein complexes with cofactors to regulate cellular cholesterol levels, primarily by facilitating cholesterol trafficking.^6^ Progesterone receptor membrane component 1 (PGRMC1) and low-density lipoprotein receptor (LDLR) bind to σ_2_R, forming a trimeric complex that modulates the uptake of cholesterol and amyloid-β_4__2_ (Aβ_4__2_).^7^ In addition, σ_2_R may also interact with Niemann-Pick C1 (NPC1), a protein essential for transporting cholesterol out of lysosomes.^8^ Emerging evidence further suggests that σ_2_R may be directly involved in cholesterol synthesis. The σ_2_R gene (TMEM97) is a transcriptional target of sterol regulatory element-binding protein 2 (SREBP-2)^9^ and appears to be regulated by steroid-dependent feedback mechanisms.^10^

The recently resolved X-ray crystal structure of bovine σ_2_R reveals that the protein forms a transmembrane homodimer, with both protomers adopting an identical conformation.^4^ Each protomer consists of 176 amino acids and is composed of four transmembrane helices, enclosing a central ligand-binding cavity. In addition to these transmembrane segments, each protomer features a short helix positioned at the top of the structure (Figure 1A). Several residues, H_21_, D_29_, D_56_, E_73_, Q_77_, T_110_, and V_146_, have been identified as critical for ligand binding (Figure 1B).^4^ Furthermore, the cocrystal structure with cholesterol suggests that σ_2_R is capable of directly recognizing sterol molecules, providing new mechanistic insights into its role in regulating cholesterol homeostasis.^4^

**Figure 1:**
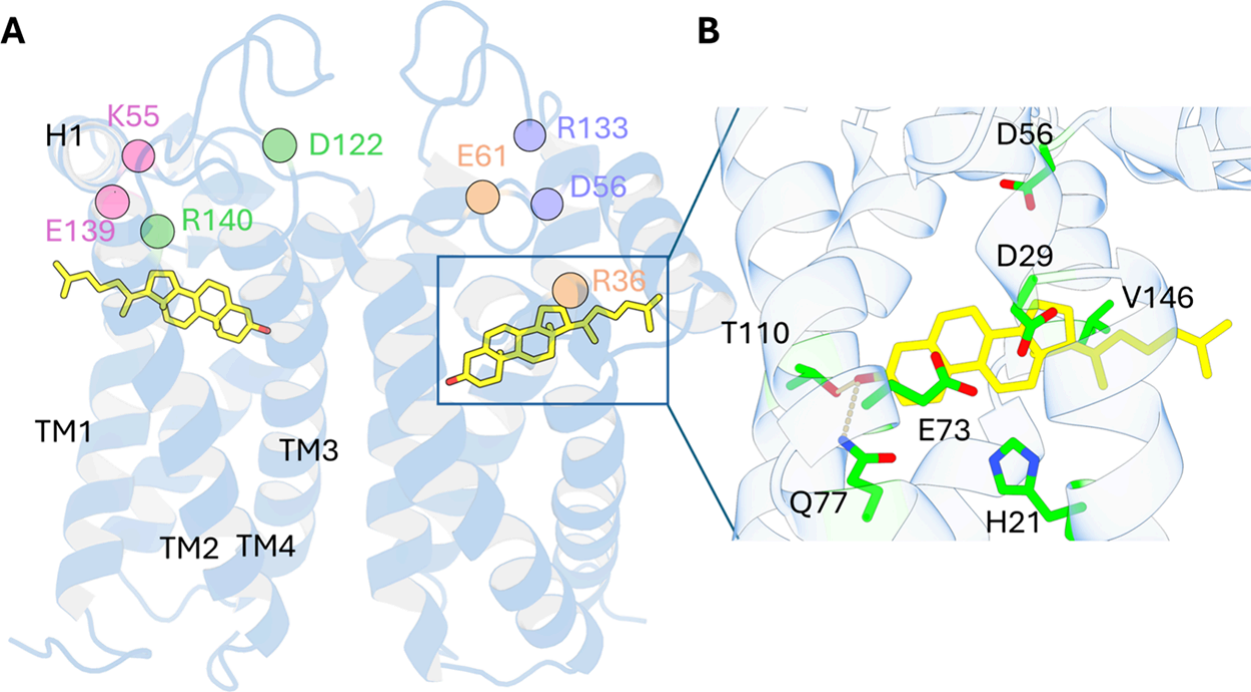
Structural features of the human σ_2_ receptor. (A) Structure of human σ_2_R generated using homology modeling based on the bovine σ_2_R crystal structure (PDB: 7MFI^4^). The spheres indicate the Cα atoms of the key residues probed in this study. (B) Close-up view of the ligand-binding pocket. The bound ligand, cholesterol, is shown in light yellow.

As a putative therapeutic target for cancer and Alzheimer’s disease, the σ_2_R has attracted significant interest in the search for selective ligands.^11^ Several ligands have demonstrated promising efficacy in preclinical tumor models,^12^ and σ_2_R selective fluorescent probes have shown strong potential in cancer diagnostics.^13^ However, despite its pharmacological importance, the molecular mechanism of σ_2_R function remains poorly understood. In contrast to σ_1_R, which undergoes ligand-induced oligomerization, the oligomeric state of σ_2_R appears to be unaffected by ligand binding,^4^ and the structural and functional roles of its dimeric assembly remain ambiguous.^4,14^ Although molecular dynamics (MD) simulations have been employed to investigate σ_2_R conformational dynamics, very few studies have examined the receptor in its dimeric form,^14,15^ leaving a significant gap in our understanding of its structural and functional regulation.

To characterize the functional motions of the dimeric receptor, we performed adaptive sampling MD simulations. Unlike traditional MD simulations that rely on a single long trajectory, adaptive sampling generates hundreds of shorter trajectories in an iterative manner.^16^ This approach helps the system escape from local energy minima and enhances the exploration of rare conformational events without introducing external bias.^16,17^ Previous studies have shown that protein dynamics contain significant anharmonic components and that anharmonicity is essential for describing biologically relevant motions.^18–21^ Regular analysis tools, usually based on second-order statistics like principal component analysis (PCA), often underestimate protein anharmonicity.^22,23^ To identify these anharmonic modes in σ_2_R, we applied Quasi-Anharmonic Analysis (QAA), which was designed to extract nonlinear modes directly from MD simulation data.^22,24,25^ While QAA predominantly focuses on global backbone motions, we quantified localized binding pocket dynamics using Convolutional Variational Autoencoder (CVAE). Since protein dynamics are inherently high-dimensional, visualizing protein dynamics landscape often depends on prior knowledge of the system.^26^ As an unsupervised learning method, autoencoders overcome this and have been widely implemented to analyze protein dynamics^26–28^ and several published studies have proved its advantages in identifying metastable states and clustering conformational ensembles.^29–33^ In this study, the combined use of QAA and CVAE allowed us to connect large-scale backbone rearrangements with local pocket fluctuations, providing a comprehensive view of the dynamics in the σ_2_R. These analyses revealed anticorrelated motions between the backbones of the two protomers in the σ_2_R homodimer, as well as asymmetric fluctuations within their active sites. The observed anticorrelation suggests that ligand binding to one protomer may allosterically reduce the likelihood of binding to the other, providing plausible insights into the dimeric role of the receptor.

## Results and Discussion

### Inter-Chain Anticorrelation in the σ2 Receptor Backbone

In the crystal structure, the ligand-binding pocket is enclosed by transmembrane helices (Figure 1A). Among these helices, the distance between TM1 and TM4 spans the entrance to the ligand-binding cavity and thus serves as a key indicator of pocket opening. To describe this motion more accurately, we computed the centroid distance between the TM1 (residues P_23_-D_29_) and TM4 (residues E_137_-Y_147_) segments in each protomer (Figure 2A). The centroid distance captures the overall movement of both helices rather than a single residue pair, providing a more representative measure of pocket conformation (Figure 2A). To distinguish the open and closed conformational states of the pocket, we defined the threshold distance based on the local minimum in the bimodal distribution of helix distances in chain B. The two peaks correspond to the closed and open states, respectively, and the local minimum (21.34 Å) was used as the cutoff separating the two states. Conformations with distances greater than this threshold were classified as open, and distances less than or equal to this threshold were classified as closed. Analysis of the joint probabilities of the four possible dimeric states in the apo systems revealed imbalance between the two protomers as shown in Table 1. Chain B is found in the open state 59.5% of the time. Consequently, the asymmetric state (A closed, B open) is 1.73 times more probable than the reverse state (A open, B closed). These findings suggest either insufficient conformational sampling or an inherent nonequivalence between the two protomers.

**Table 1:** Joint Conformation Frequency of the σ_2_R Homodimer (Apo)

conformation	frequency
both closed	21.30%
both open	26.28%
A closed/B open	33.22%
A open/B closed	19.20%

**Figure 2:**
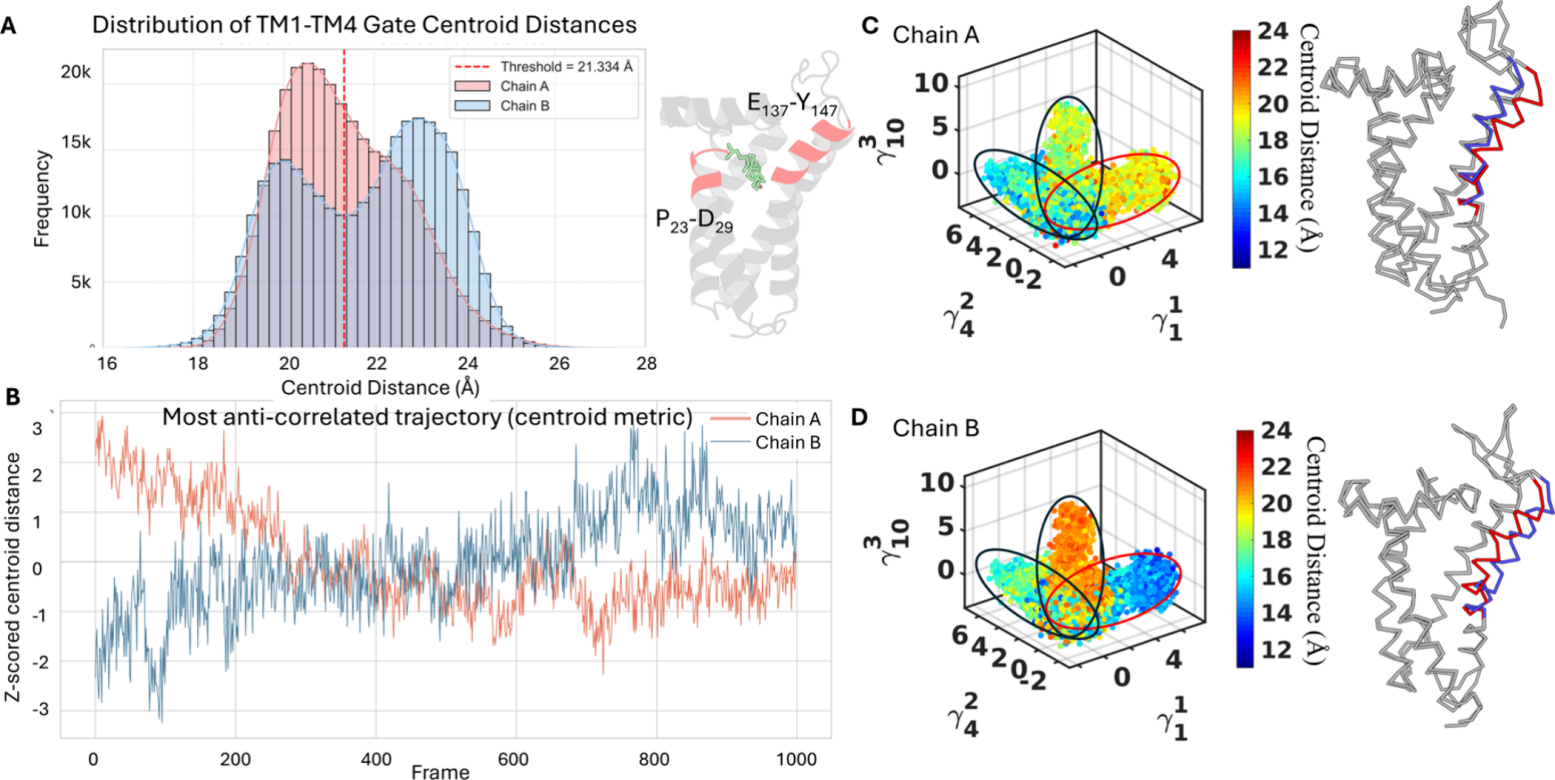
Quasi-anharmonic analysis of the σ_2_R backbone. (A) Distribution of the gate centroid distance in chain A and chain B, representing the range of pocket conformations sampled in the apo state. The threshold distinguishing open and closed states was defined as the local minimum (21.34 Å) between the two peaks in the bimodal distance distribution of chain B. (B) Trajectory showing the strongest anticorrelation (*r* = −0.6) between the two chains. (C, D) Anharmonic conformational landscape colored by gate centroid distance in chain A and chain B, with the γ_1_ anharmonic mode shown. The blue structure represents the initial conformation, while the red structure depicts the final conformation along the identified anharmonic transition.

To examine the relationship between the helical movements of the two protomers, we calculated the correlation between their TM1-TM4 gate centroid distances across all adaptive sampling trajectories. Each trajectory was treated independently to avoid artifacts arising from discontinuous data. Overall, no consistent strong correlation was observed across the entire data set (*r* = −0.02 ± 0.21), which is expected given the independent nature of adaptive sampling simulations (Figure S1). However, several individual trajectories exhibited pronounced anticorrelated motions between the two protomers (Figure 2B).

Quasi-Anharmonic Analysis (QAA) was performed to determine whether the transient anticorrelated motions reflect a functionally relevant collective motion rather than random, nonfunctional fluctuations. Anharmonicity in conformational fluctuations is a key characteristic of biologically functional dynamics, as rare, non-Gaussian motions often correspond to large-scale transitions between metastable states.^18–21^ The most significant anharmonic mode γ_1_^1^ corresponds to an anticorrelated motion of the two protomers, in which chain A adopts a more open conformation while chain B remains closed (Figure 2C,D). In this mode, the TM4 helix of chain A shifts outward, opening the binding pocket, while the corresponding TM4 in chain B moves inward, closing its pocket. In contrast, the tenth motion (γ_10_^3^) displays the opposite arrangement, although the magnitude of this motion is notably smaller, with chain B in the open state and chain A closed (Figure 2C,D). Despite these two modes, other anharmonic modes (like γ_4_^2^) capture local rearrangements that are not directly related to pocket gating, with no significant differences observed between the two protomers (Figure S2). The strong correspondence between the gating distance and the leading anharmonic mode suggests that the asymmetric alternation between open and closed protomer states represents the principal low-frequency collective motion underlying σ_2_R dynamics.

### Asymmetric Dynamics in the σ2 Receptor Apo Binding Pocket

In addition to the observed anticorrelated backbone motions, the binding pockets of the σ_2_R homodimer also display asymmetric local dynamics. To probe this in greater detail, we employed a convolutional variational autoencoder (CVAE) to learn high-dimensional representations of local structural variability within the binding pocket. The CVAE was trained in an unsupervised manner using pairwise distance matrices derived from molecular dynamics trajectories, without supplying chain identity or temporal labels. The resulting latent space was visualized using PaCMAP,^34^ revealing substantial overlap between the embedded distributions of chain A and chain B (Figure S3A). This overlap reflects a shared global conformational landscape, consistent with the expected structural symmetry of the homodimer. However, a closer examination of the local density within this space revealed distinct differences. Each chain exhibited preferential sampling of specific conformational states (Figure 3A). These chain-specific preferences suggest that, despite sharing access to similar conformational states, chain A and chain B display asymmetric dynamic behavior in the apo form.

**Figure 3:**
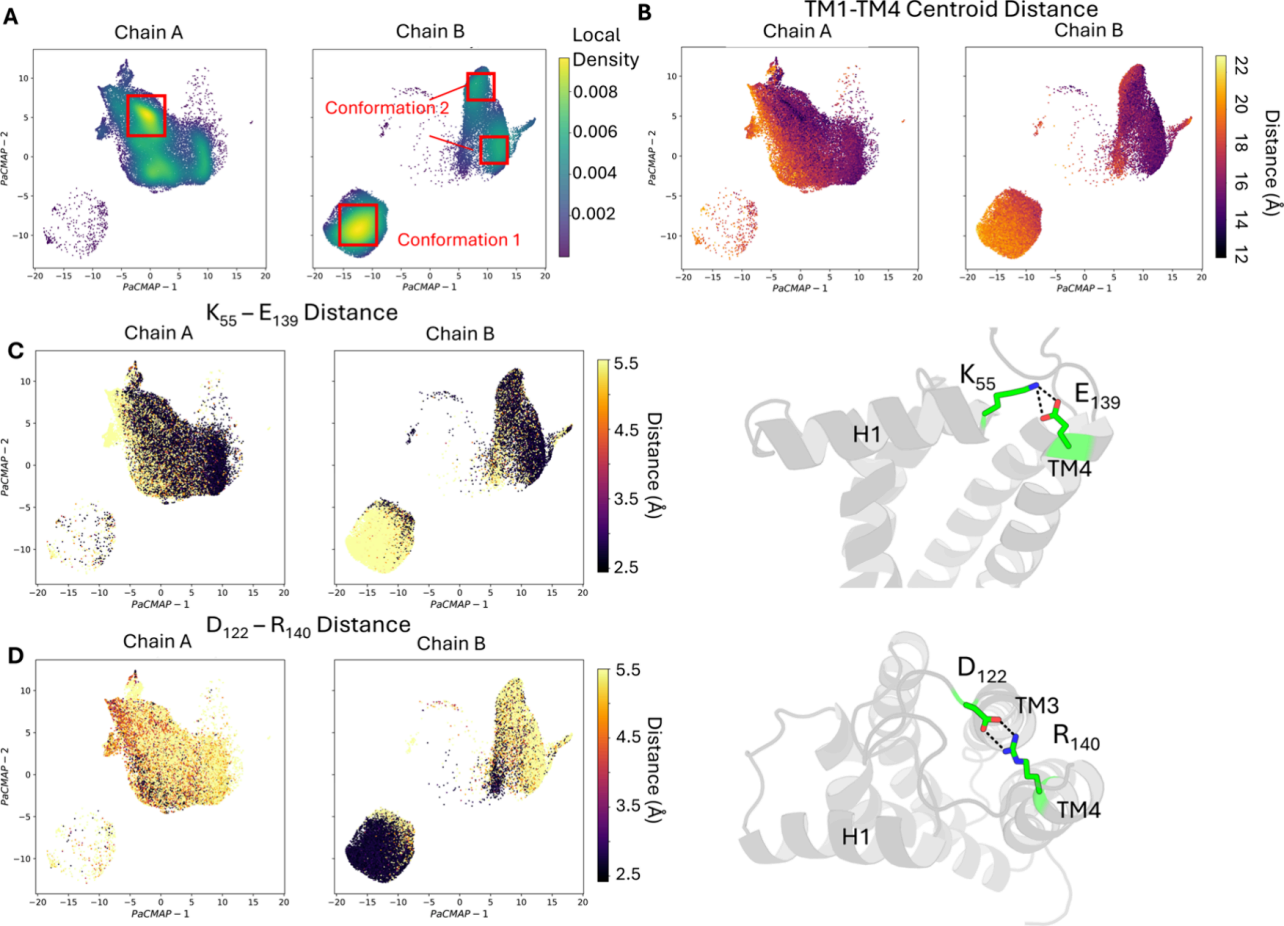
Asymmetric conformational preferences and salt bridge dynamics in the apo σ_2_ receptor. (A) Local density plots of chain A and chain B projected onto the CVAE-derived conformational space. (B) Conformational space colored by the gate centroid distance, representing the separation between TM1 and TM4. (C) Conformational space colored by the minimum distance between all potential donor–acceptor atom pairs of residues K_55_ and E_139_, reflecting the formation of the H1–TM4 salt bridge. (D) Conformational space colored by the minimum distance between all potential donor or acceptor atoms of residues D_122_ and R_140_, corresponding to the TM4–loop interaction.

To further investigate the conformational asymmetry between the two protomers, we again used the gate centroid distance as a metric to color the CVAE-derived conformational space (Figure 3B). A distinct conformational cluster located in the lower-left region, which is sampled predominantly by chain B, exhibited a relatively large gate centroid distance, consistent with a more open pocket conformation. In contrast, chain A showed minimal occupancy in this region, suggesting restricted sampling of this open state. Thus, chain B conformation 1 corresponds to the open state, while chain A and chain B conformation 2 represent the closed state. We therefore refer to these as the open and closed conformations, respectively.

To understand why chain B more frequently adopts the open-pocket conformation compared to chain A, we examined molecular interactions that may stabilize or destabilize specific conformational states. Representative conformations were extracted from the energy minima (i.e., highest-density regions) of both chain A and chain B in the latent space (Figure 3A and Figure S4). Detailed interaction analysis revealed two salt bridges that undergo significant reorganization between conformations. The first, K_55_–E_139_, linking helix H1 and TM4, was highly populated in both chain A (68.5%) and in chain B closed conformation (84.3%), but was nearly absent in chain B open conformation (Tables S1–S3). Conversely, the second salt bridge D_122_–R_140_, connecting TM4 to the TM3–TM4 loop, increased in frequency from 8.4% (chain A) and 14.9% (chain B) in the closed conformation to 92.6% in the open conformation (Tables S1–S3). Chi-squared analysis comparing the combined closed state versus the open state confirmed that the alteration in both K_55_–E_139_ and D_122_–R_140_ occupancies are highly significant (*p* < 0.001), rigorously confirming the reorganization across the conformational change (Table S6).

Visualization of the latent space colored by the presence of each salt bridge (Figure 3C,D) revealed a mutually exclusive pattern between the two interactions, suggesting a compensatory relationship. Specifically, the disruption of the K_55_–E_139_ salt bridge appears to release TM4 from its anchored position, while the formation of D_122_–R_140_ may help reposition TM4, promoting a shift toward the open-pocket conformation. These findings suggest that a salt bridge rewiring mechanism may drive the conformational transition associated with pocket opening in chain B and may explain the asymmetric sampling behavior observed in the σ_2_R homodimer.

### Asymmetric Dynamics in the σ2 Receptor Binding Profile

To investigate whether the asymmetric dynamics observed in the apo state persist during ligand binding, we next analyzed molecular dynamics simulations of the cholesterol-bound σ_2_R system (holo). We defined ligand occupancy based on the center-of-mass distance between the cholesterol molecule and the pocket, using a threshold of 7.5 Å, which is approximately half the length of cholesterol (∼15.5 Å). As illustrated in Figure 4A, the homodimer exhibits a markedly asymmetric cholesterol binding profile between its two protomers. Ligand occupancy analysis revealed that 63.02% of the trajectory features single-chain binding events, with 62.35% of those involving chain B. Only 4.72% of the trajectory exhibited simultaneous occupancy of both chains (Table 2), suggesting that the receptor strongly favors a one chain occupied conformation when interacting with cholesterol.

**Table 2:** Distribution of Ligand Occupancy States across the Simulation Trajectory

ligand binding state	frequency
chain A occupied	0.67%
chain B occupied	62.35%
both chains occupied	4.72%
both chains unoccupied (apo)	32.36%

**Figure 4:**
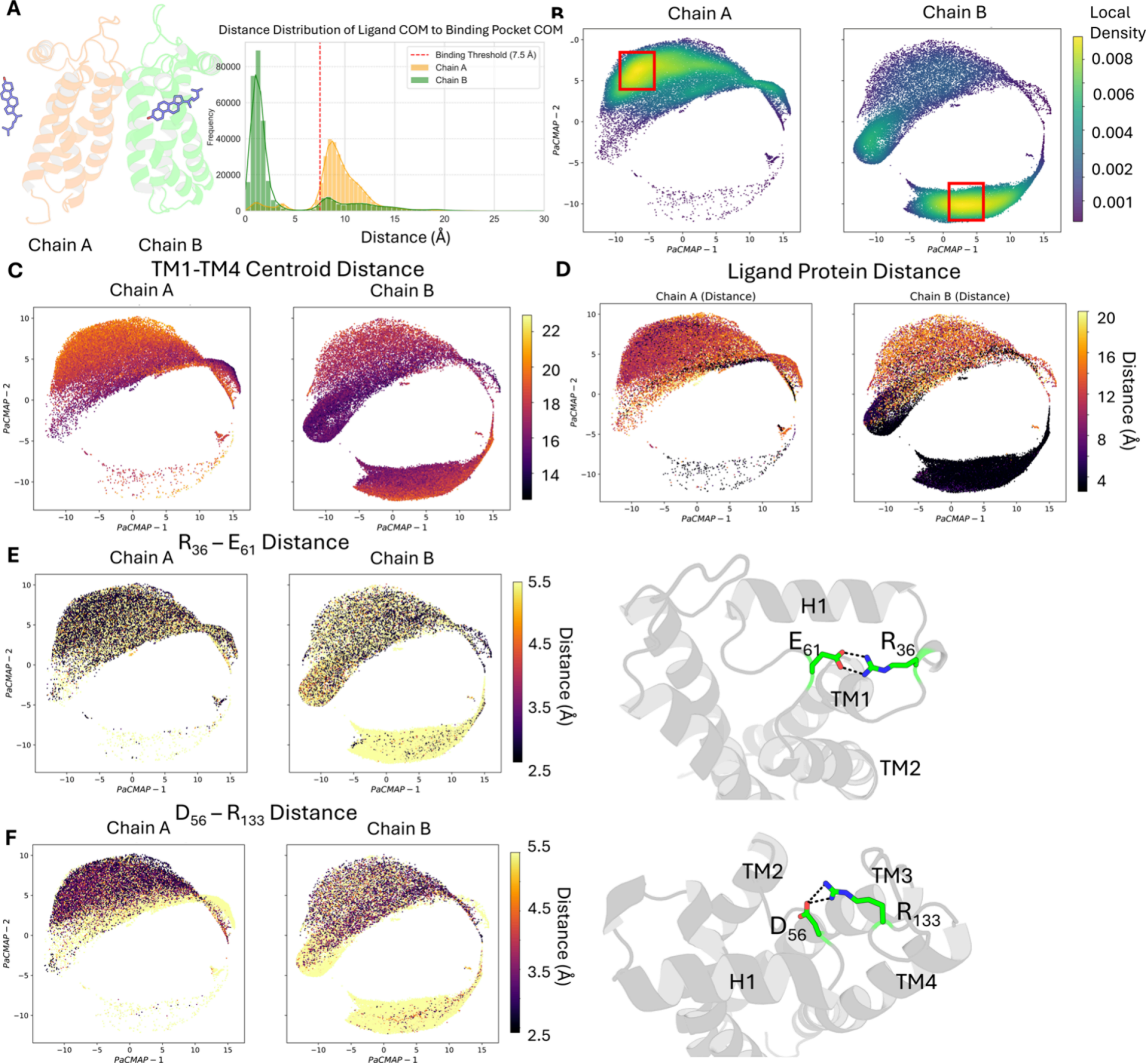
Conformational landscape and salt bridge rearrangement in the cholesterol-bound σ_2_R. (A) Cholesterol distance distribution to σ2R binding pocket. (B) Local density plots of chain A and chain B projected onto the conformational space. (C) Conformational space colored by the gate centroid distance, representing the separation between TM1 and TM4. (D) Conformational space colored by the ligand–protein distance, defined as the minimum atomic distance between Q_77_ (side-chain nitrogen) and cholesterol (oxygen atom). (E) Conformational space colored by the minimum distance between all potential donor–acceptor atom pairs of residues R_36_ and E_61_. (F) Conformational space colored by the minimum distance between all potential donor–acceptor atom pairs of residues D_56_ and R_133_.

Similar joint probabilities for the four possible dimeric states was calculated (Table 3). We now defined the open/closed threshold as the mean TM1–TM4 gate centroid distance derived from ligand-occupied conformations (21.34 Å), ensuring the closed state corresponds to conformations stabilized by ligand occupancy. The analysis shows that the asymmetric A open B closed conformation is relatively populated (32.32%). In the presence of cholesterol, chain B slightly prefers the closed conformation (57.62%).

**Table 3:** Joint Conformation Frequencies of the σ_2_R Homodimer (Holo)

conformation	frequency
both closed	25.30%
both open	26.06%
A closed/B open	16.32%
A open/B closed	32.32%

Consistent with the apo system, CVAE analysis of the cholesterol-bound σ_2_R revealed that chain A and chain B share a similar overall conformational space (Figure S3B) yet differ in their occupancy preferences (Figure 4B). When colored by the gate centroid distance, chain A was found to adopt a relatively more open conformation compared to chain B (Figure 4C). This is consistent with the ligand occupancy data, which showed that chain B is predominantly cholesterol-bound throughout the simulation, implying a more closed pocket conformation. To further investigate the relationship between ligand binding and pocket dynamics, we colored the conformational space by the distance between the ligand and the protein, calculated as the atomic distance between the side chain nitrogen of Q_77_ and the oxygen atom of the bound cholesterol (Figure 1B). The resulting distribution suggests a clear association between TM1–TM4 separation and ligand binding, where conformational states with wider gate centroid distances are more likely to exhibit ligand dissociation in both chains (Figure 4D).

Representative structures were extracted from the highest-density regions of the conformational space (Figure 4B) to further explore conformational differences in the holo system (Figure S5). The salt bridge K_55_–E_139_, which was associated with the closed state in the apo simulations, remained highly occupied in the closed conformation of chain B in the holo system (93.9%). Interestingly, this salt bridge was also present at a moderate frequency (65.3%) in the open conformation of chain A (Tables S4 and S5), suggesting that cholesterol binding in one protomer may allosterically influence the dynamics of the other, and that chain A in the bound system does not fully recapitulate the apo open state. In contrast, the D_122_–R_140_ salt bridge, which previously associated with TM4 flexibility and pocket opening in the apo system, was largely absent in both chains of the cholesterol-bound receptor (Tables S4 and S5). This salt bridge, which connects TM4 to the TM3–TM4 loop, was hypothesized to facilitate TM4 movement and enable pocket expansion. Its disruption in the bound system may reflect the stabilizing influence of cholesterol. As a highly hydrophobic ligand, cholesterol likely reinforces compact pocket conformations by restricting TM4 flexibility. These findings suggest that cholesterol binding to one chain not only stabilizes a closed conformation in that protomer but also alters the dynamic potential of the unbound chain, disrupting the salt-bridge switching mechanism observed in the apo state.

In addition to the previously characterized interactions, cholesterol binding was associated with the rearrangement of two newly identified salt bridges: R_36_–E_61_ and D_56_–R_133_. The R_36_–E_61_ interaction connects two loop regions, which are between helix H1 and TM1 and between H1 and TM2, respectively. In the apo system, this salt bridge was observed in 40–60% of the extracted conformations. However, in the holo system, while its frequency remained similar in chain A, the interaction was completely lost in cholesterol-bound chain B, suggesting a strong association between ligand binding and disruption of this salt bridge (Figure 4E). Given its location near the binding site, the breaking of R_36_–E_61_ may play a role in modulating the position of H1 and thus stabilizing the cholesterol within the pocket.

The second salt bridge, D_56_–R_133_, links the two longest loops within the receptor, the H1–TM2 loop and the TM3–TM4 loop. This interaction was not present in either protomer of the apo system, nor in chain B of the holo system. However, it formed with high frequency (79.8%) in chain A of the holo system (Figure 4F). This pattern suggests that D_56_–R_133_ is not disrupted by cholesterol binding but instead emerges as an allosteric response in the unbound protomer. The formation of this interaction highlights a potential long-range coupling mechanism between the two protomers, reinforcing the idea that ligand binding induces asymmetric and cooperative structural rearrangements within the σ_2_R homodimer. All significant alterations in salt bridge occupancy were confirmed using Chi-squared analysis (all *p* < 0.001, Table S6).

To better understand the functional implications of these rearrangements, we quantitatively analyzed the dynamic coupling among the key salt bridges and the ligand–pocket distance using a correlation matrix (Figure 5). Consistent with the structural analysis, K_55_–E_139_ showed a mild but positive correlation with ligand binding, supporting its role as a closed states associated interaction that is modulated rather than directly triggered by cholesterol engagement. In contrast, the D_122_–R_140_ salt bridge, which previously acted as the primary open-state switch in the apo receptor, exhibited negligible correlation with ligand binding in the holo system. This apparent independence likely arises because cholesterol binding quenches the conformational flexibility of TM4, locking the bridge in its disrupted state. In this scenario, the loss of D_122_–R_140_ is better interpreted as a consequence of ligand binding, reflecting a downstream structural stabilization.

**Figure 5:**
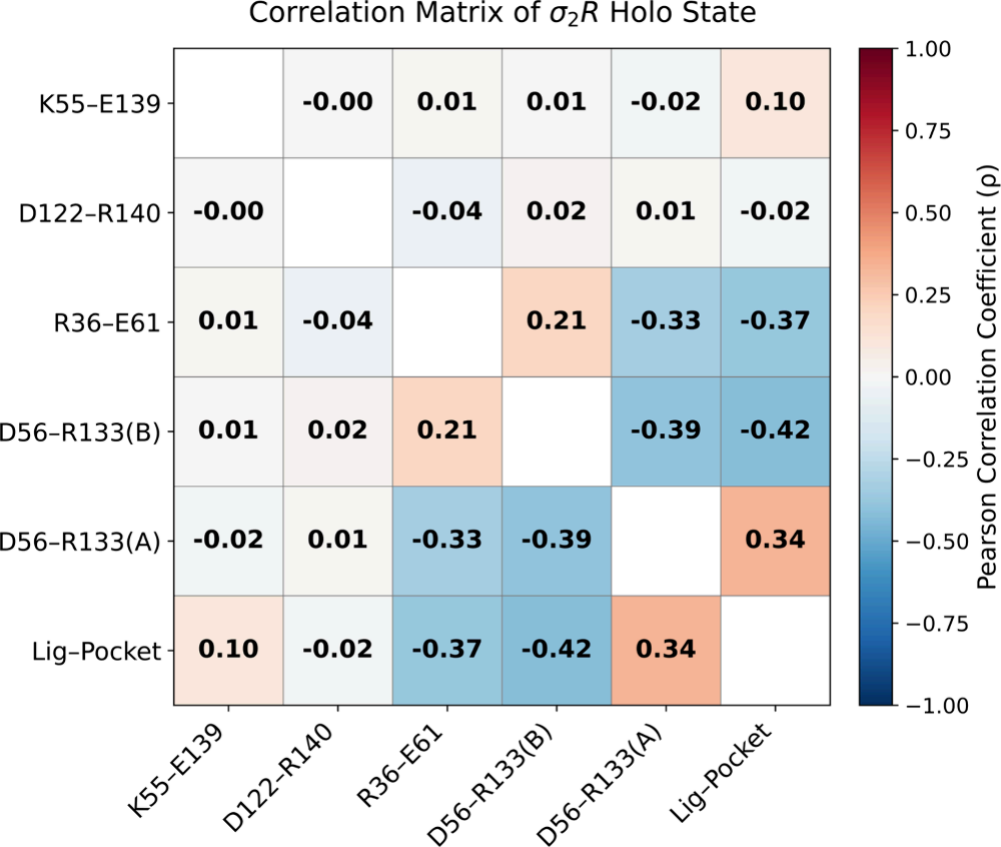
Correlation matrix of the σ_2_R holo state showing dynamic relationships between the distances of key salt bridges and the ligand–pocket interaction. Salt-bridge distances were calculated as the minimum distance between oppositely charged atoms, while the ligand–pocket distance was defined between the side-chain nitrogen atom of Q_77_ and the oxygen atom of the bound cholesterol.

The two newly identified interactions, R_36_–E_61_ and D_56_–R_133_ (B), displayed apparent negative correlations with ligand binding, indicating that they are disrupted upon ligand engagement. Interestingly, the correlation analysis reveals a positively associated relationship between D_56_–R_133_ in chain A and the ligand–pocket interaction in chain B, providing further evidence for a potential allosteric coupling between the two protomers.

### Validation of Ionic Switches

To validate the functional roles of the proposed ionic switches derived from our human σ_2_R MD simulations, we conducted a detailed structural comparison with the bovine σ_2_R crystal structure (PDB ID: 7MFI^4^). The crystal structure provides strong experimental support for the asymmetric behaviors observed in our simulations. Both protomers bind cholesterol, yet the K_55_–E_139_ salt bridge is present in only one protomer and absent in the other (Figure 6A). This asymmetric pattern is consistent with our observation that the K_55_–E_139_ interaction is associated with pocket closure, while ligand occupancy in one protomer can alter the conformation of its partner chain. The presence of this asymmetric interaction in the crystal structure suggests that, even though both protomers are ligand-bound and crystallized, their intrinsic dynamics may still be asymmetric.

**Figure 6:**
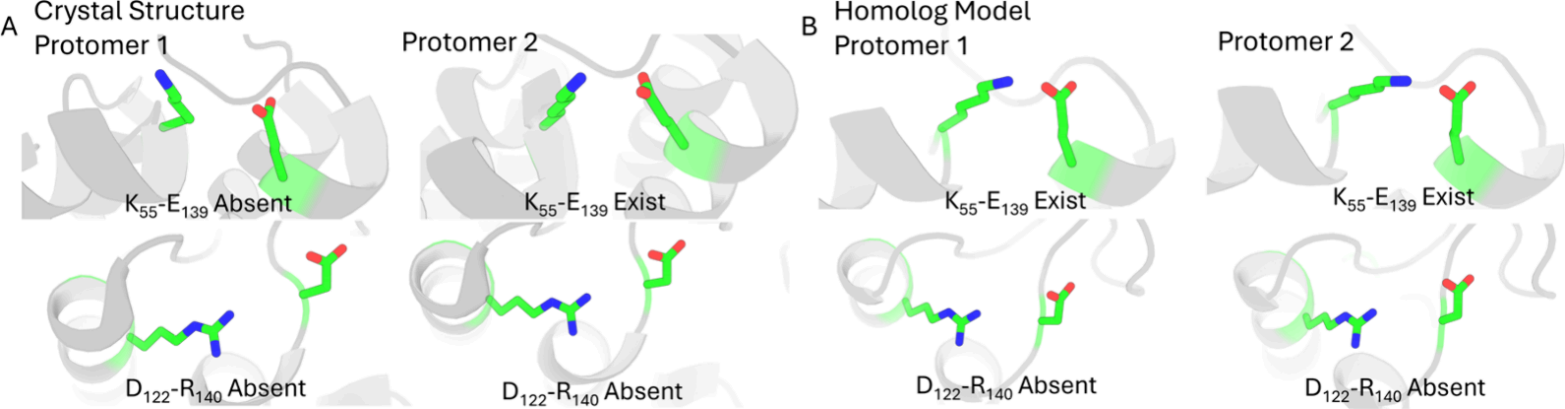
Comparison of key ionic interactions between the σ_2_R crystal structure (PDB ID: 7MFI^4^) and the human σ_2_R homology model.

Conversely, the D_122_–R_140_ bridge was identified associated with open states in our simulations. In agreement with this assignment, the D_122_–R_140_ interaction is lost or significantly weakened in the ligand-bound, closed-like protomer of the crystal structure (Figure 6A). This supports the MD-derived mechanism in which ligand induced closure structurally requires the disruption of this salt bridge. Importantly, although our homology model was built symmetrically, with both protomers initially containing the K_55_–E_139_ interaction and lacking the D_122_–R_140_ bridge, the simulations spontaneously developed asymmetric conformations (Figure 6B). This confirms that the preference for single-chain pocket closure was not an artifact of the initial model setup.

Two additional ligand-binding–induced salt bridges observed in the human σ_2_R simulations, R_36_–E_61_ and D_56_–R_133_, are absent in the bovine structure due to sequence differences (Figure S6). This species specificity suggests that the allosteric effect involving D_56_–R_133_ is unique to the human receptor and underscores the importance of using human σ_2_R models to fully capture the electrostatic network relevant to its regulation.

### Asymmetric Lipid–Protein Interaction Upon Binding to Cholesterol

To examine whether membrane interactions contribute to the asymmetric binding behavior of the σ_2_R homodimer, we quantified protein–lipid contacts throughout the simulation trajectories. In the apo system, lipid contacts were symmetrically distributed between the two protomers, indicating that the asymmetric backbone motions observed in the absence of ligand were not driven by differences in membrane interactions (Figure 7A). In contrast, the holo system exhibited increased lipid contact in chain B, specifically along transmembrane helices TM2 and TM3 (Figure 7B,C). This enhanced interaction was accompanied by increased structural stability in the adjacent loop connecting TM3 and TM4, as shown in Figure 7D. These findings suggest that cholesterol binding may indirectly modulate membrane interactions in one protomer, contributing to localized stabilization of specific structural elements and reinforcing the asymmetric dynamic behavior of the homodimer.

**Figure 7:**
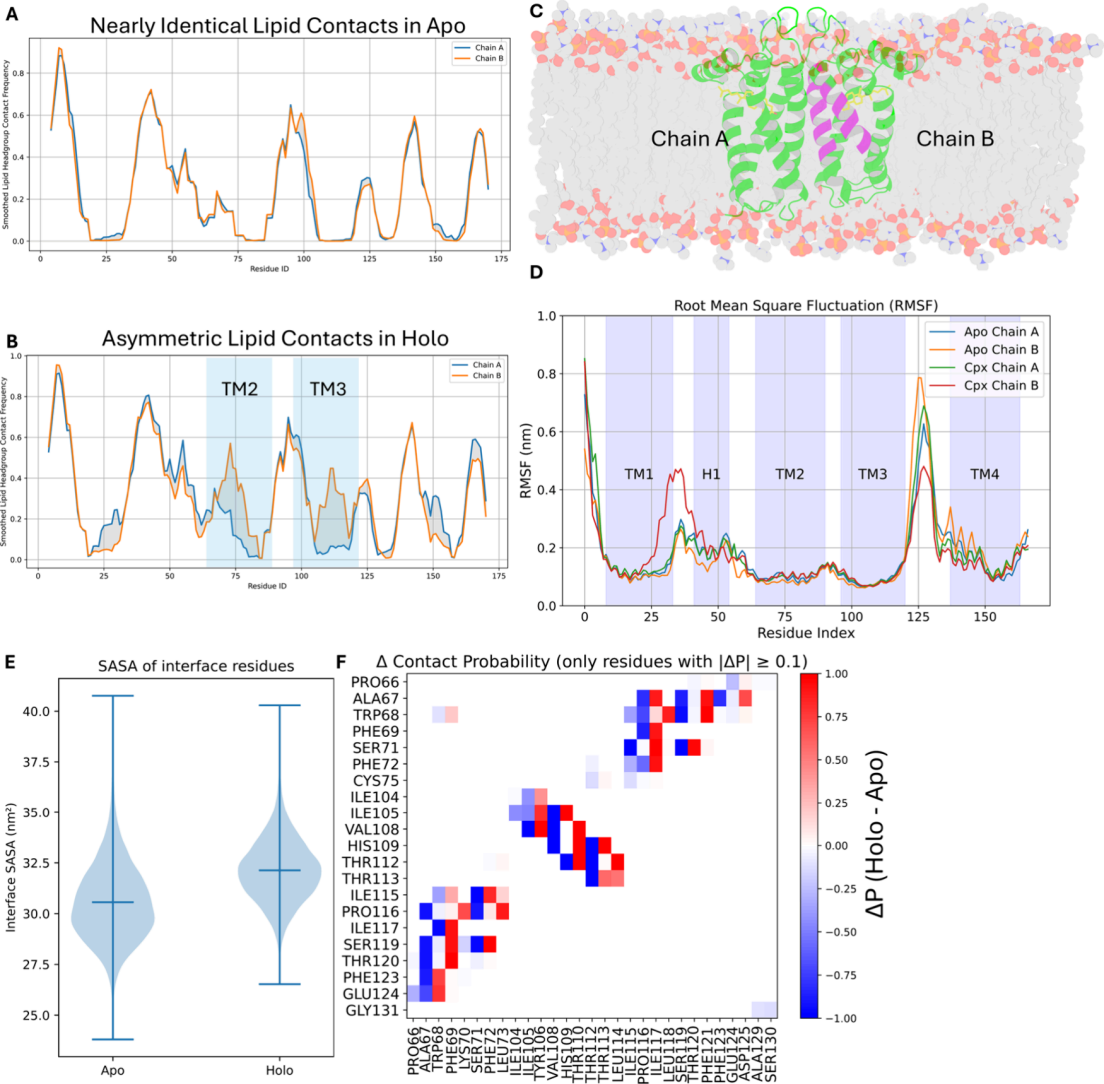
Lipid–protein interactions and structural stability in the apo and cholesterol-bound (holo) σ_2_ receptor systems. (A, B) Smoothed lipid headgroup contact frequency plotted across protein residues for the apo system (A) and the holo system (B). (C) Protein buried within the lipid membrane, with purple highlights indicating areas showing increased lipid–protein contacts in the holo state. (D) Root mean square fluctuation (RMSF) profiles for each chain in both systems. (E) Solvent-accessible surface area (SASA) values for interface residues. (F) Residue–residue contact map showing regions with increased (red) or decreased (blue) contact probability between the apo and holo states, using a threshold of |Δ*P*| ≥ 0.1.

We analyzed the dimeric interface to evaluate whether ligand binding transmits allosteric effects between the two protomers. The solvent-accessible surface area (SASA) of the interface was calculated to assess potential conformational rearrangements upon ligand binding (Figure 7E). Only a mild increase in interfacial SASA was observed in the holo system, suggesting that ligand binding slightly perturbs the transmembrane helix packing, leading to a minimal expansion toward the solvent. Furthermore, residue–residue contact maps were computed to identify regions exhibiting notable changes in interprotomer interactions (Figure 7F). The contact probability differences appeared to be locally compensatory without any specific region showing a concerted gain or loss. These results indicate that the dimeric interface remains largely stable upon ligand binding, and the structural perturbation is modest, consistent with a limited degree of interprotomer allosteric communication.

### Insights Behind Asymmetry

Asymmetry is not an uncommon phenomenon in dimeric transmembrane receptors. For instance, MD simulations of homodimeric rhodopsin, a member of G-protein coupled receptors (GPCRs), revealed unequal structure changed between the protomers, suggesting asymmetric activation in symmetric dimers.^35^ Cryo-EM structure analysis reveals structurally asymmetric conformation for Pendrin, a homodimeric ion-transporter,^36^ and ATP-binding cassette (ABC) transporters.^37^

The asymmetry observed in these dimers have provided plausible explanations for their dimerization or oligomerization. First, an allosteric effect within the dimer allows one protomer to transmit signal while the partner remains poised, increasing dynamics range or sensitivity for signaling.^38^ Second, asymmetry may enable selective partnering or recruitment of downstream effectors. For example, one protomer could preferentially bind a G-protein or β-arrestin, while the other binds regulatory or scaffold proteins, thereby integrating multiple inputs. Finally, membrane-embedded dimers may exploit asymmetric dimer interfaces to modulate ligand binding, protomer-protomer crosstalk and receptor activation kinetics.^39^ For instance, molecular-dynamics and interface-mapping studies show that lipids and membrane environment can bias one protomer’s conformation over the other.^40^

The asymmetric dynamics of σ_2_R were first hypothesized as a plausible explanation for the bell-shaped dose–response curves commonly observed for σ-receptor ligands.^41,42^ The crystal structure further supports this notion by showing that, under high local ligand concentrations, both protomers can bind the ligand simultaneously, indicating that occupancy of one site does not sterically preclude binding at the other. This asymmetric binding behavior could therefore conceptually account for the biphasic response. However, due to the limited receptor selectivity of many early σ-ligands, this effect could not be conclusively attributed to σ_2_R. Although several σ_2_R-selective ligands have since been reported to display similar bell-shaped responses,^43^ interpretation remains complicated by the fact that σ_2_R lacks intrinsic catalytic activity and that most experiments were conducted in cellular or *in vivo* systems, where off-target effects cannot be fully excluded.

Combined with asymmetric dynamics observations in other transmembrane dimers, we proposed plausible explanations for the dimeric nature of the σ_2_R. σ_2_R likely functions as part of a higher-order protein complex.^7^ Thus, rather than accommodating a second ligand, the partner protomer may preferentially engage with associated proteins such as PGRMC1 or LDLR within the TMEM97–PGRMC1–LDLR complex, thereby coupling ligand recognition to downstream signaling or trafficking events. This model aligns with current views that dynamic asymmetry enables one protomer to act as an allosteric modulator or adaptor for protein–protein interactions, while the other serves as the principal ligand-binding or signaling unit.

## Conclusions

The σ_2_R is a pharmacologically important membrane protein involved in cholesterol regulation and overexpressed in cancer and neurological disorders. Despite increasing interest, detailed mechanistic understanding of its functional dynamics has remained limited. In this study, we combined adaptive sampling molecular dynamics simulations with quasi-anharmonic analysis and unsupervised machine learning to investigate the structural behavior of σ_2_R in both apo and cholesterol-bound (holo) states. Our results reveal a striking asymmetry within the σ_2_R homodimer. This asymmetry is driven by anticorrelated backbone motions and state-specific salt bridge rearrangements, including a mutually exclusive switching between K_55_–E_139_ and D_122_–R_140_. Upon cholesterol binding, one protomer adopts a stabilized closed state, while the unbound protomer undergoes altered dynamics and salt bridge reorganization. This asymmetric behavior, together with the receptor’s known association with other cofactors, provides a plausible rationale for the dimeric architecture of σ_2_R. The receptor appears to preferentially maintain a single-protomer-occupied conformation, in which the unbound chain may facilitate downstream interactions with partner proteins. Notably, the species-specific allosteric salt bridge D_56_–R_133_ may play a critical role in modulating human σ_2_R function, highlighting the necessity of future mutagenesis studies to validate its mechanistic contribution.

While our findings reveal a clear mechanistic basis for asymmetric protomer behavior and sterol-induced conformational modulation, it is important to acknowledge the limitations associated with using cholesterol as the model ligand. The functional role of cholesterol as a σ_2_R ligand remains uncertain, and the sterol densities observed in the template bovine crystal structure were tentatively assigned.^4^ Subsequent study has proposed 20­(S)-hydroxycholesterol as a more potent endogenous ligand,^44^ yet its structural similarity to cholesterol and much lower cellular abundance suggest that cholesterol binding may still hold physiological relevance, particularly if σ_2_R functions as a sterol-sensing or scaffolding protein. Therefore, we do not expect that the asymmetric behavior observed in our simulations arises specifically from cholesterol binding but rather reflects an intrinsic property of the σ_2_R dimeric architecture and its general mode of sterol recognition.

## Methods

### System Preparation and Molecular Dynamics Simulation

A homology model of the human σ2R dimer was built using the bovine σ2R (PDB ID: 7MFI^4^) as a template, which shares 78% sequence identity. The modeling was performed using Modeler v10.5.^45^ Sequence alignment between human and bovine σ2R was carried out using Espript3 ^46^ (Figure S6), followed by model optimization and validation with PROCHECK.^47^ This approach was chosen as it retains the precise binding site geometry and cholesterol-interacting residues observed in the bovine template, ensuring functional relevance for subsequent studies. The initial model was cleaned by removing nonessential molecules, retaining only the cholesterol molecules bound in the active site. Two systems were prepared for simulation: a cholesterol-bound complex (Holo) and ligand-free form (Apo). The initial binding poses for the cholesterol molecules in the active site were determined by structural alignment of the homology model to the 7MFI crystal structure using PyMOL.^48^

System preparation was performed with the HTMD Suite.^49^ The titratable residues protonation states were predicted at pH 7 for both systems using the PROPKA 3.0 ^50^ and PDB 2PQR tools^51^ implemented within the protein prepare function.^52^ Each system was embedded in a mixed lipid bilayer composed of a 1-palmitoyl-2-oleoylphosphatidylcholine (POPC):Cholesterol (CHL) mixture at an 80:20 ratio, constructed with the HTMD membrane builder and sized at 120 × 120Å^2^, guided by the protein’s orientation from the OPM database.^53^ For the Holo system, two cholesterol molecules were initially placed at the identified binding sites with orientation based on the crystal structure, resulting in a 1:2 protein–ligand stoichiometry. The parameters from the CHARMM36m force field^54^ were applied for the protein, lipids, and ions and the TIP3P water model^55^ was used as solvent. The systems were solvated with a 20 Å layer of water using the solvate­() function. Following this, the charmm.build­() function was used to neutralize the systems with 0.15 M NaCl and complete the system building, preparing them for the equilibration phase. Each system was equilibrated under NPT conditions at 310 K for 40 ns using ACEMD^56^ as the MD engine. Prior to equilibration, the systems underwent 5000 steps of energy minimization using the conjugate gradient (CG) algorithm. The default options were kept the same for the rest of the settings as provided by HTMD.

For each system, multiple short simulations were performed using an adaptive sampling strategy based on reinforcement learning. More specifically, the AdaptiveBandit enhanced sampling protocol^57^ was used that employs the Upper Confidence Bound (UCB1) algorithm which selects states that have high uncertainty (under sampled) or have high estimated reward (underexplored regions). The first epoch started with 4 simulations run for 100 ns. At the end of the epoch, the trajectories are pooled, and the conformational space discretized into a Markov State Model (MSM), with the free energy being estimated from the stationary distribution of each state. The next epoch starts from a state that are either poorly sampled or expected to yield high information gain, with an exploration value of 0.01 and a goal function of 0.3. After each adaptive sampling round, trajectories are initiated with randomized initial velocities, ensuring sufficient sampling diversity without the need for separate replicate simulations. The MetricSelfDistance function evaluated native Cα contacts across residues to build the MSMs. Simulations were carried out using the ACEMD molecular dynamics engine^56,58^ under an NVT ensemble with a Langevin thermostat (damping coefficient of 0.1 ps^–1^). A hydrogen mass repartitioning scheme was applied to enable a 4 fs integration time step. Trajectory frames were recorded every 0.1 ns during production runs. Following this, convergence was evaluated based on the Cα RMSD (Figure S7). A summary of all generated trajectories is provided in Table 4.

**Table 4:** Overview of Simulation Trajectories

system	number of trajectories (×100 ns)	total simulation time (μs)
apo	304	30.4
holo (cholesterol bound)	334	33.4

### Z-Score Normalization

To enable direct comparison of helical distances between σ_2_R protomers, we applied z-score normalization. Z-score normalization removes differences in absolute scale by centering each distance value around its mean and scaling by its standard deviation.^59^ For a given time point *X* the normalized value *z* is defined as

$$z=\frac{X-\mu }{\sigma }$$ 1

where μ is the mean and σ is the standard deviation of the original time series.

To quantify the relationship, we calculated the Pearson correlation coefficient, *r*, defined as the covariance of the two series divided by the product of their standard deviations.^60^ An *r* value of +1 indicates perfect correlation, −1 indicates perfect anticorrelation, and 0 indicates no linear correlation.

### Quasi-Anharmonic Analysis (QAA)

Traditional approaches like MSM, which are used to analyze protein dynamics, dependent on linear dimension reduction methods like principal component analysis (PCA) and time-lagged independent component analysis (tICA). While effective at capturing dominant motions, these methods are limited to second-order statistics and primarily detect linear correlations in the data. As a result, they tend to underestimate the anharmonic and nonlinear behavior of real-life protein dynamics. Functionally relevant motions, which are often rare, subtle, and localized, are typically governed by higher-order correlations and long-tailed fluctuation distributions.^61^ Linear techniques struggle to resolve these features, highlighting the need for analytical frameworks that incorporate higher-order statistics to better characterize the complex energy landscapes of proteins.^62^

In our study, we utilized a high ordered analyzing method (QAA) to give a comprehensive insight into the dynamics of the σ2R. QAA uses fourth-order statistics, which allows a better description of the anharmonic and non-Gaussian fluctuations.^62^ We provided the Cartesian coordinates of all Cα atoms as input, and the method returned the top 10 anharmonic modes ranked by statistical significance. To interpret the motions in a biologically meaningful way, trajectories projected along each mode were colored according to gate centroid distance, a metric that reflects the open–closed transitions of the ligand-binding pocket. All ten modes were examined, and the most significant mode (γ_1_) was identified as associated with asymmetric gate opening and closure. Mode γ_10_ was manually selected as a representative inverse motion of γ_1_, while γ_4_ was included as an example of an anharmonic mode unrelated to pocket gating, serving as a reference for local conformational rearrangements. These visualizations enabled identification of anharmonic processes associated with pocket dynamics and interprotomer asymmetry.

### Convolutional Variational Auto Encoder (CVAE)

A convolutional variational autoencoder (CVAE) was used to capture and cluster the conformational dynamics of the ligand-binding pocket for each protomer in the σ_2_R homodimer. The CVAE architecture combines convolutional layers with variational inference to learn compact latent representations from high-dimensional distance maps, enabling unsupervised identification of subtle local conformational states.^28^

To define the input space, residues within 5 Å of the bound cholesterol molecule in the crystal structure were selected, yielding a consistent set of 27 active-site residues. The same residue set was used for both apo and holo systems across both protomers. These include: H_21_, I_24_, T_25_, M_28_, D_29_, L_46_, L_47_, W_49_, Y_50_, F_54_, D_56_, L_59_, F_66_, F_69_, L_70_, E_73_, Q_77_, F_81_, Y_103_, T_107_, T_110_, L_111_, I_114_, V_146_, Y_147_, P_149_, and Y_150_. For each chain, a 27 × 27 pairwise Cα distance matrix was computed per frame. Both systems were processed independently using the same CVAE architecture and training protocol. The model included two convolutional layers (64 filters each) with kernel size 1, stride 1, and padding 1, followed by a single dense layer with 16 units. Dropout was not applied. Training was performed for 100 epochs with a batch size of 750 and a test split of 20%. Models were trained without shuffling or early stopping.

Multiple latent dimensions were tested during training, and loss values across different dimensions were found to be highly similar (Figure S8A,B). Therefore, the final latent dimension was selected based on the clarity of latent space visualization. Model quality was validated by comparing decoded distance matrices to the original inputs to ensure structural features were preserved (Figure S8C,D). Embeddings were labeled according to chain identity and key inter-residue distances for downstream visualization and correlation analysis.

### Trajectory Extraction and Interaction Detection

Representative conformations were selected from the highest-density regions of the latent space for each chain. Binding interactions were analyzed using MDAnalysis.^63,64^ We evaluated hydrogen bonds and salt bridges using a distance cutoff of 4 Å. Although π–π and cation−π interactions were also screened using standard geometric criteria, no significant differences were observed across chains, and these interactions were not further considered. Significant chain-specific hydrogen bonds and salt bridges were identified and compared. Their interatomic distances were computed and mapped onto the conformational embeddings to facilitate structural interpretation and visualization.

### Lipid–Protein Contact Calculation

To quantify the frequency of contacts between protein residues and lipid headgroups, we performed a distance-based contact analysis using MDAnalysis.^63,64^ Lipid headgroup atoms were defined as phosphorus (P), nitrogen (N), and oxygen atoms (O11, O12) of POPC, along with the O3 atom of CHL1. For each frame in the trajectory, the minimum distance between all atoms in a given protein residue and all selected lipid headgroup atoms was computed. A contact was defined if at least one interatomic distance between a residue and the lipid headgroups was below a 6.0 Å cutoff. The number of contact-forming frames was recorded for each residue across the entire trajectory and normalized by the total number of frames to obtain a contact frequency. To facilitate interpretation, the per-residue contact frequency profiles for each chain were smoothed using a moving average with a window size of 7 residues. The resulting profiles represent the likelihood of persistent lipid interactions along the protein sequence and were used for downstream visualization and interpretation of membrane-binding hotspots.

## Supplementary Material



## Data Availability

The data sets generated and/or analyzed during the current study, including molecular dynamics simulation setup files for the human sigma-2 receptor (σ_2_R) homodimer and representative conformations extracted from the CVAE latent space, are available at 10.5281/zenodo.16881219.

## Notes

Salt bridge occupancy in the apo system; top 10 salt bridges in the apo system; salt bridge occupancy in the holo system; top 10 salt bridges in the holo system; statistical validation of key salt bridge occupancies; gate centroid distance between protomers; anharmonic modes; conformational space overlap; representative conformations from the apo system; representative conformations from the holo system; sequence comparison between bovine and human sigma-2 receptors; rmsd per frame; CVAE statistics (PDF)
