# Supplementary material for: Asymmetric Dynamics Between the Protomers of the σ2 Receptor Homodimer
Source: J Chem Inf Model. 2025 Nov 6;65(22):12511–23. doi: 10.1021/acs.jcim.5c02174 (PMC12648649; doi:10.1021/acs.jcim.5c02174)
Supplement: Supplementary file 1 [file ci5c02174_si_001.pdf]

Supporting Information

Asymmetric Dynamics Between the Protomers of the σ2 Receptor Homodimer

Manming Xu,[1] Saleh Alyemni,[1] Veniamin A. Borin,[2] Ranabir Majumder,[2] Nathaniel V Nucci,[3,4] Thomas Keck,[3,5] Kevin Frankowski,[6] Pratul K. Agarwal,[2,7], Shozeb Haider,[1,8,9^]

[1] UCL School of Pharmacy, London WC1N 1AX UK, UK

[2] Department of Physiological Sciences, Oklahoma State University, Stillwater, OK 74078, USA

[3] Department of Biological and Biomedical Sciences, Rowan University, Glassboro, NJ, 08028, USA

[4] Department of Physics and Astronomy, Rowan University, Glassboro, NJ, 08028, USA

[5] Department of Chemistry and Biochemistry, Rowan University, Glassboro, NJ, 08028, USA

[6] Center for Integrative Chemical Biology and Drug Discovery, Eshelman School of Pharmacy, University of North Carolina, Chapel Hill, NC 27599

[7] High-Performance Computing Center, Oklahoma State University, Stillwater, OK 74078, USA

[8] University of Tabuk (PFSCBR), Tabuk, 71491, Saudi Arabia

[9] UCL Centre for Advanced Research Computing, London WC1H 9RL, UK

^ To whom correspondence should be addressed:

Prof Shozeb Haider (Shozeb.haider@ucl.ac.uk)

Table S1 – Salt bridge occupancy in the Apo system. Top 10 salt bridges ranked by frequency in the extracted apo system chain A conformations (477 frames).

Pair	Frame Count	Frequency (%)
R8-E11	460	96.436
K55-E139	327	68.553
K125-E135	215	45.073
K52-E53	204	42.767
E37-R44	199	41.719
E61-R133	184	38.574
R36-E61	146	30.608
K130-E135	82	17.191
R36-E37	65	13.627
D122-R140	40	8.386

Table S2 – Top 10 salt bridges ranked by frequency in the extracted apo system chain B conformation 1 (271 frames).

Pair	Frame Count	Frequency (%)
D122-R140	251	92.620
R8-E11	248	91.513
R36-E61	185	68.266
K130-E135	118	43.542
K52-E53	114	42.066
E37-R44	96	35.424
E121-K125	28	10.332
E61-K125	22	8.118
D122-K125	14	5.166
E121-R133	13	4.797

Table S3 – Top 10 salt bridges ranked by frequency in the extracted apo system chain B conformation 2 (452 frames).

Pair	Frame Count	Frequency (%)
R8-E11	432	95.575
K55-E139	380	84.071
E121-R133	201	44.469
E37-R44	194	42.920
R36-E61	187	41.372
K52-E53	125	27.699
K125-E135	112	24.779
K55-E135	76	16.814
D122-R140	67	14.823
K130-E135	63	13.938

Table S4 – Salt bridge occupancy in the Holo system. Top 10 salt bridges ranked by frequency in the extracted holo system chain A conformations (277 frames).

Pair	Frame Count	Frequency (%)
R8-E11	238	85.921
D56-R133	221	79.783
K55-E139	181	65.343
R36-E61	180	64.982
K125-E135	113	40.794
E37-R44	113	40.794
D122-R140	106	38.267
K52-E53	69	24.910
D122-K125	30	10.830
E53-K130	30	10.830

Table S5 – Top 10 salt bridges ranked by frequency in the extracted holo system chain B conformations (264 frames).

Pair	Frame Count	Frequency (%)
R8-E11	256	96.970
K55-E139	248	93.939
D29-K67	97	36.742
K52-E53	84	31.818
D122-K125	63	23.864
E37-R44	60	22.727
K130-E135	52	19.697
K55-E135	52	19.697
R133-E135	48	18.182
E61-R133	36	13.636

Table S6 – Statistical validation of key salt bridge occupancies

Salt Bridge	Group 1	Group 2	χ^2	P-value	Biological Context
			**Apo System**		
**K55–E139**	Apo Closed	Apo Open	500.59	< 0.001	Loss of interaction upon pocket opening.
**D122–R140**	Apo Closed	Apo Open	655.36	< 0.001	Strong increase in interaction upon pocket opening.
			**Holo System**		
**K55–E139**	Holo Chain B	Holo Chain A	65.60	< 0.001	Significant difference suggests Chain A does not fully close.
**D122–R140**	Apo Closed	Holo	387.89	< 0.001	Interaction is lost/disrupted upon cholesterol binding.
**R36–E61**	Apo	Holo Chain B	174.48	< 0.001	Interaction is completely disrupted by ligand binding.
**D56–R133**	Apo	Holo Chain A	1119.6	< 0.001	New interaction emerges as an allosteric response in unbound protomer.

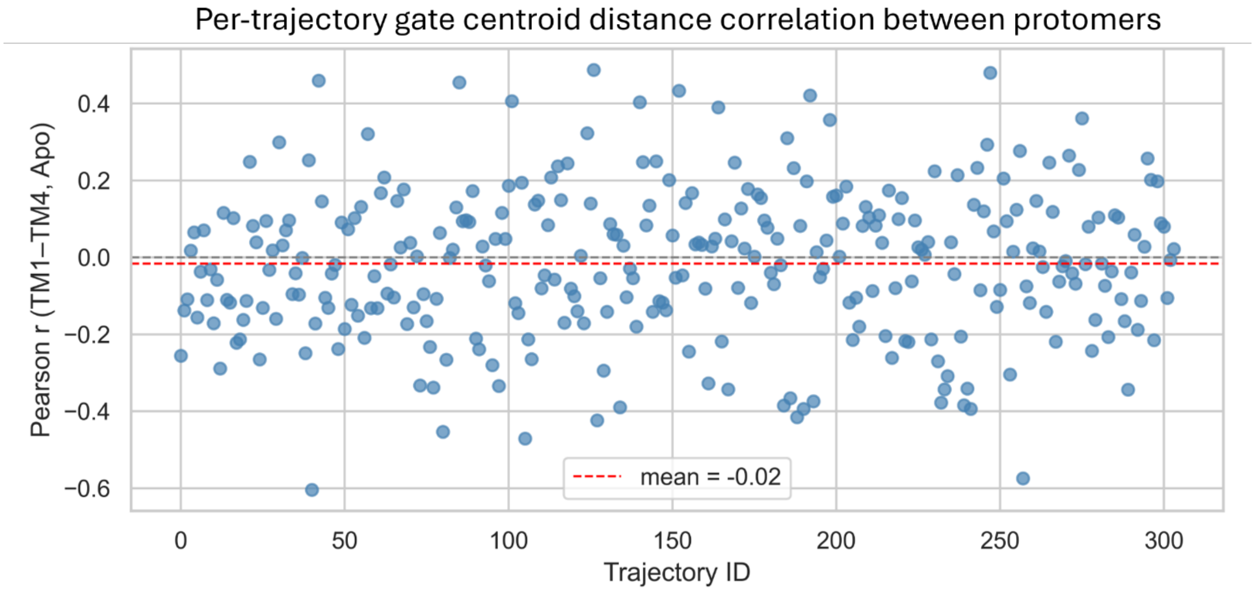

Figure S1 – Per-trajectory gate centroid distance correlation between protomers. The correlation coefficient r has a mean value of -0.02, indicating no strong correlation across all trajectories.

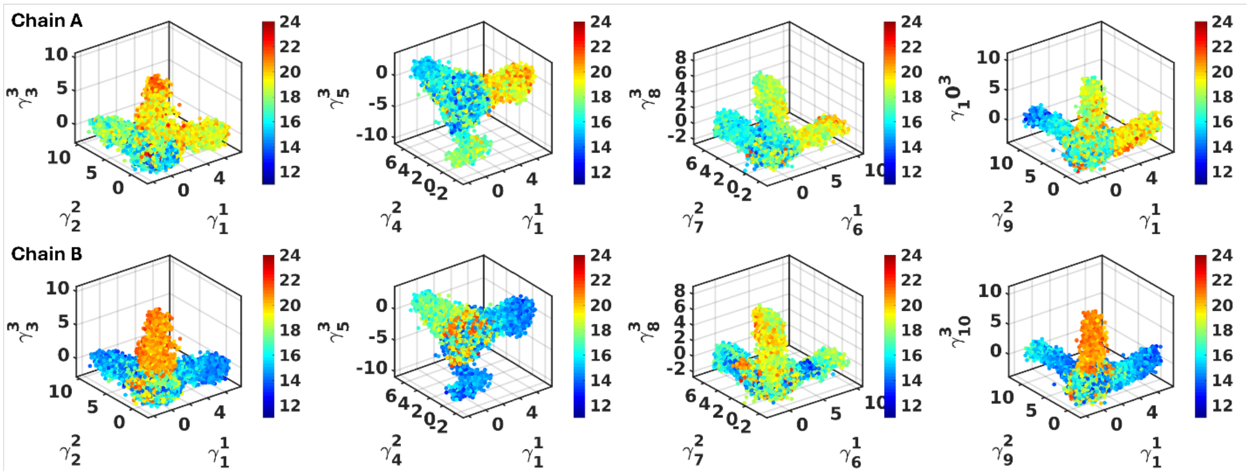

Figure S2 – All anharmonic modes detected by QAA.

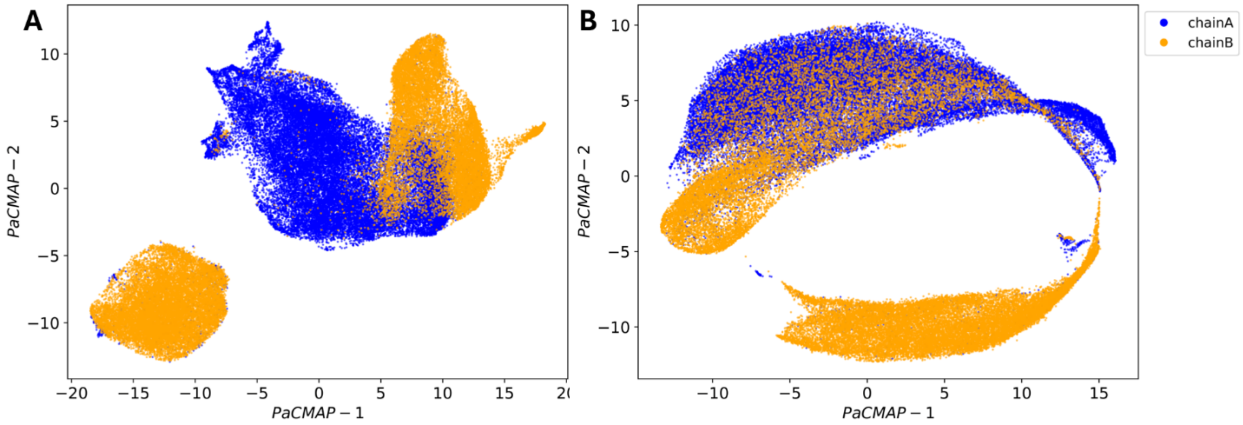

Figure S3 – A-B. Conformational space overlap between chain A and chain B in the Apo (A) and Holo (B) states.

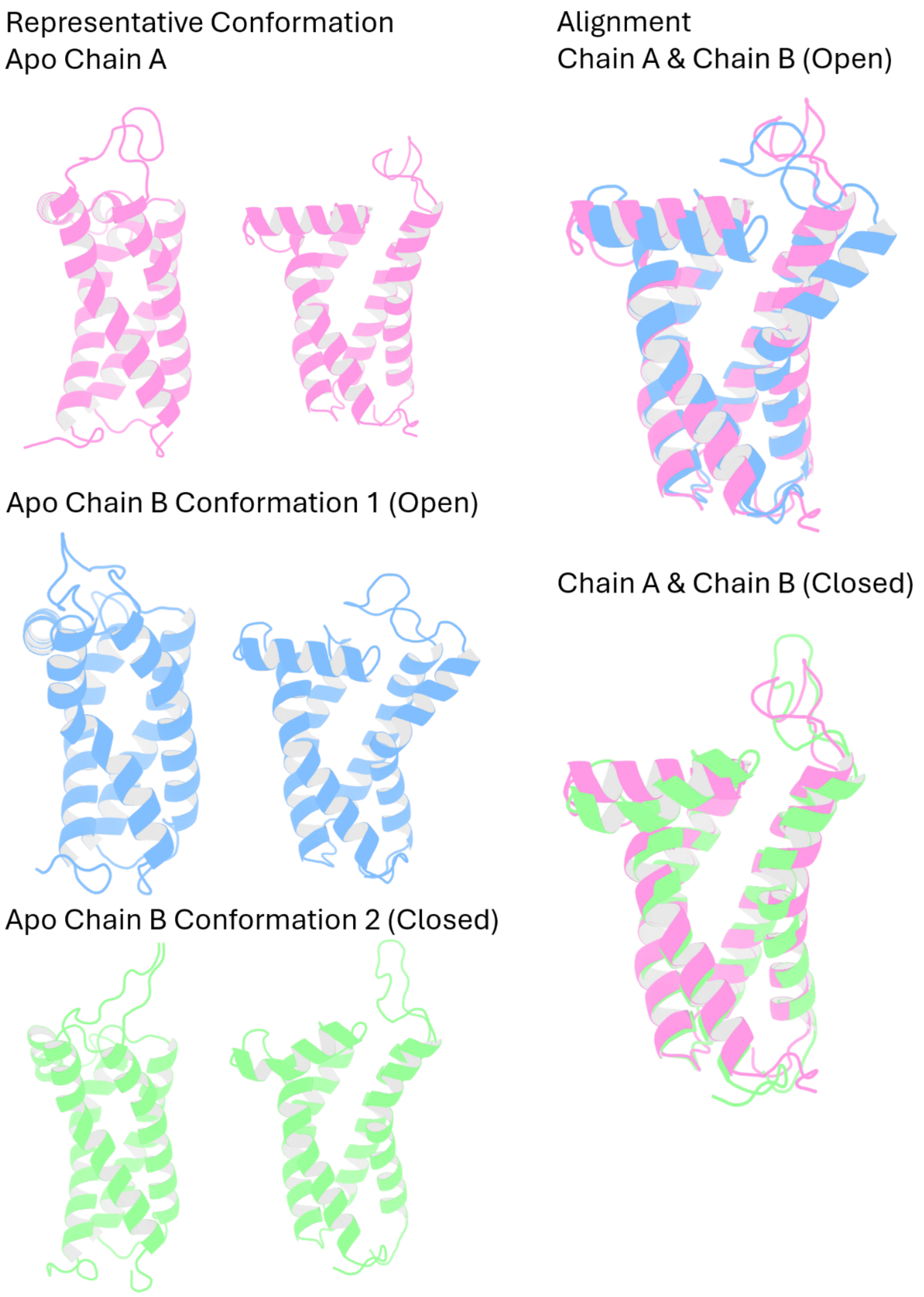

Figure S4 – Extracted representative conformation for chain A and chain B in the apo system and structure alignment.

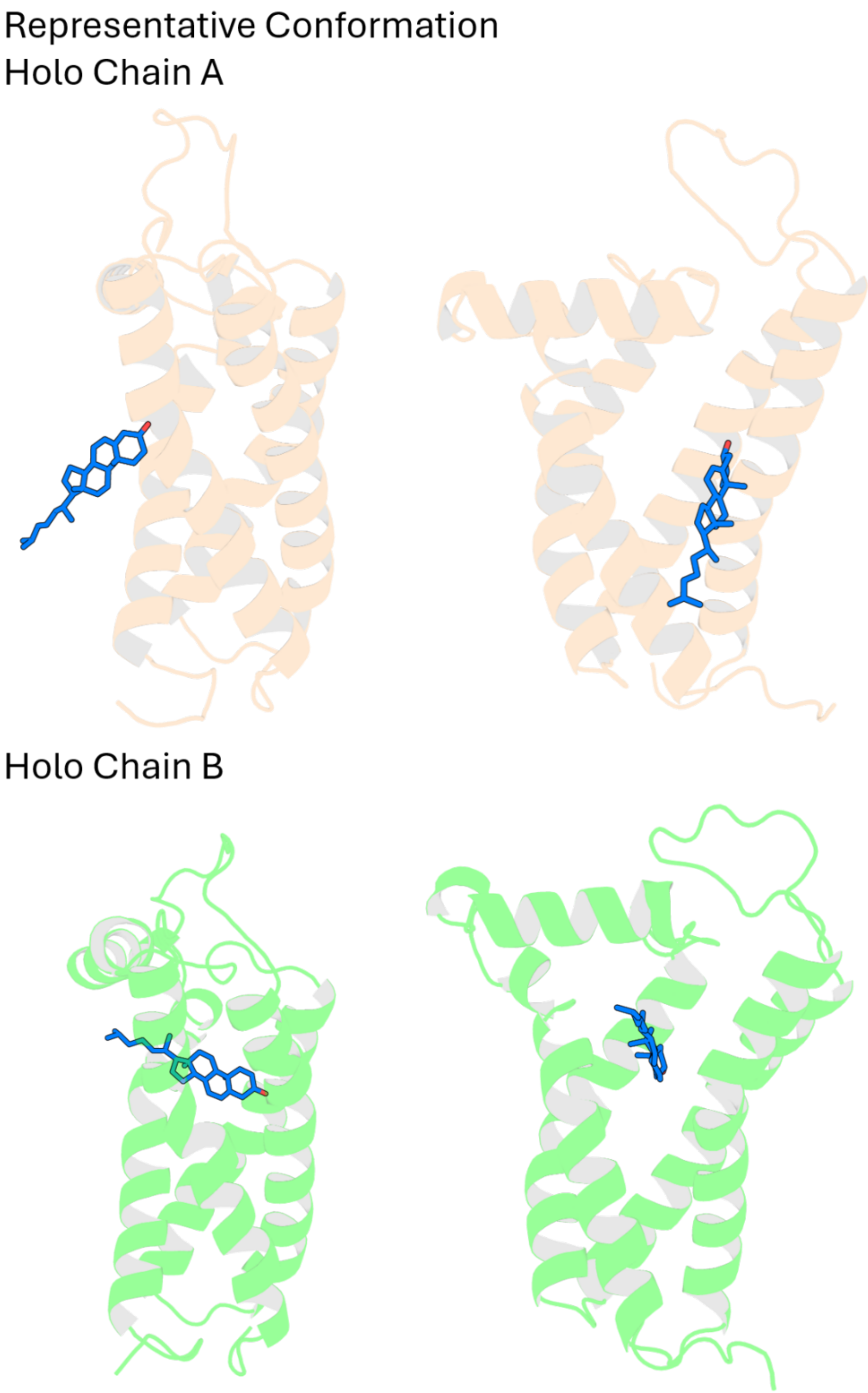

Figure S5 – Extracted representative conformation for chain A and chain B in the holo system

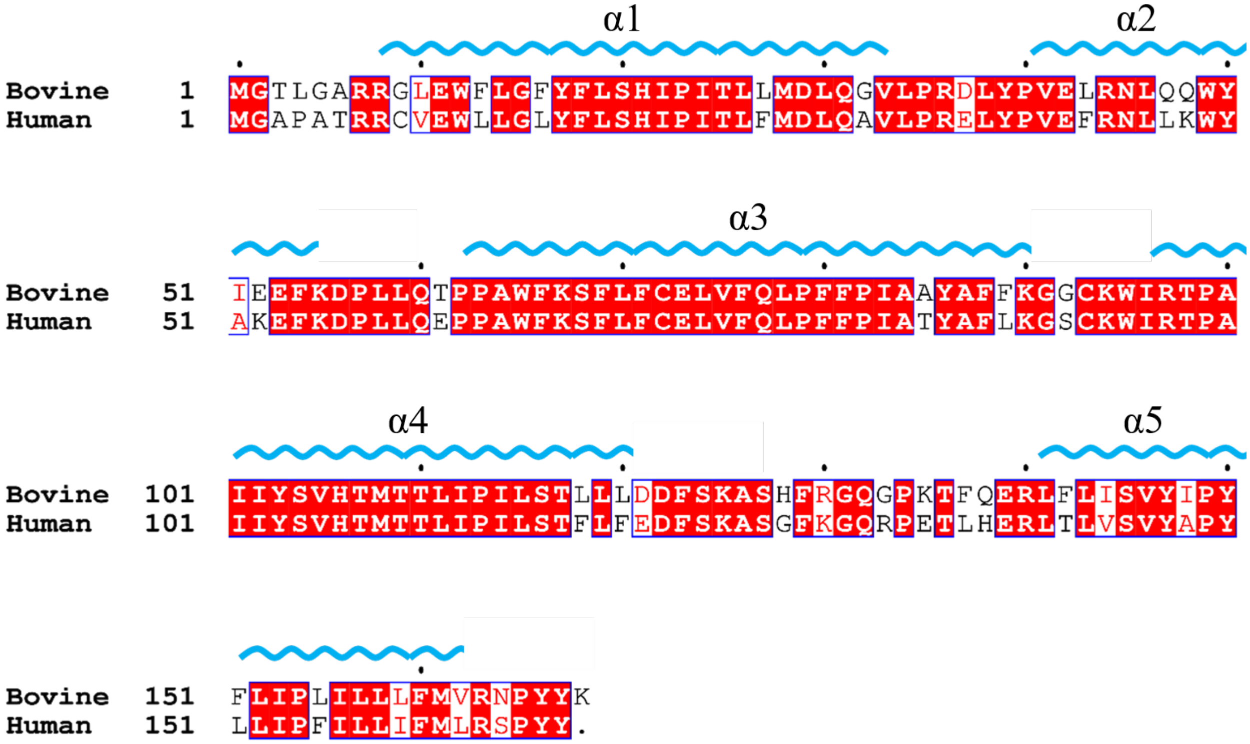

Figure S6 – Sequence alignment of bovine and human sigma-2 receptors. The proteins share a sequence similarity of 78%.

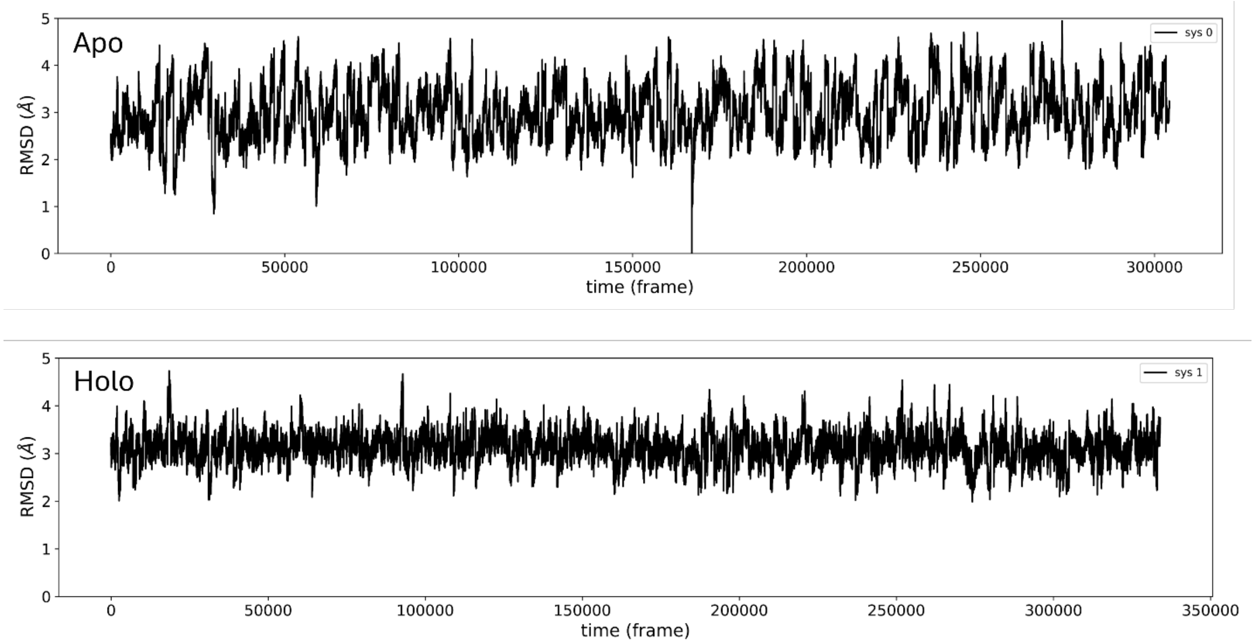

Figure S7 – Root Mean Square Deviation Per Frame for Apo and Holo System.

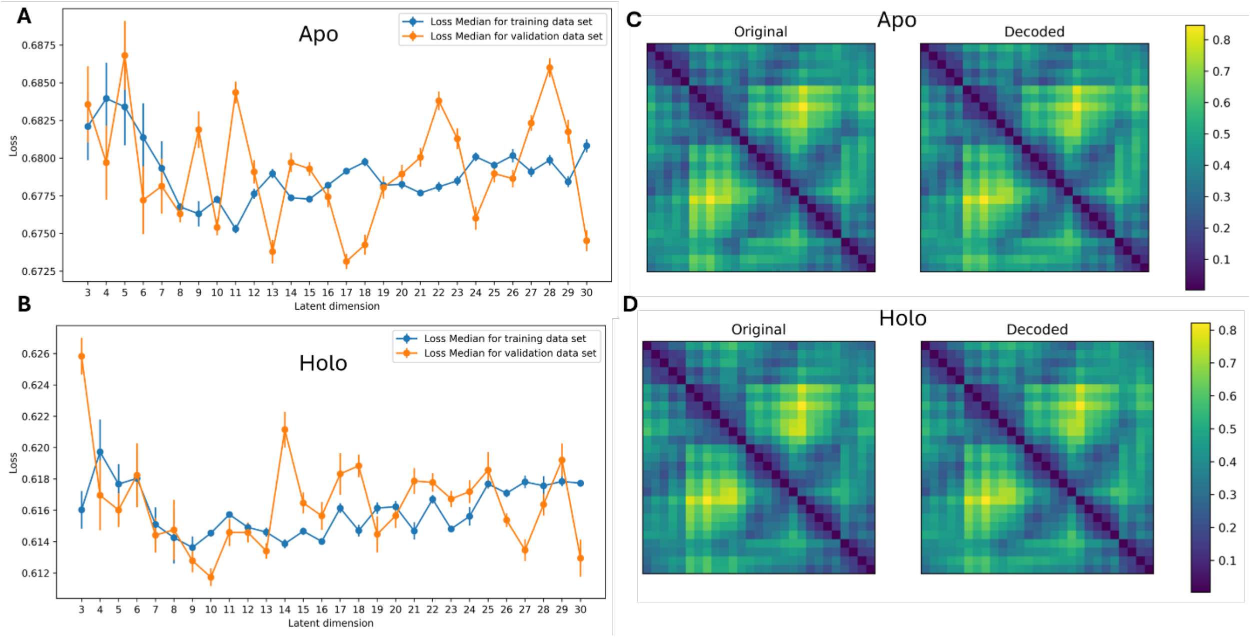

Figure S8 – A-B. Median training and validation loss during CVAE training for the apo and holo systems. C-D. Comparison between original and decoded input features, demonstrating accurate reconstruction.
